# Beliefs and Values About Music in Early Childhood Education and Care: Perspectives From Practitioners

**DOI:** 10.3389/fpsyg.2019.00724

**Published:** 2019-04-24

**Authors:** Margaret S. Barrett, Libby M. Flynn, Joanne E. Brown, Graham F. Welch

**Affiliations:** ^1^School of Music, The University of Queensland, Brisbane, QLD, Australia; ^2^School of Psychology, The University of Queensland, Brisbane, QLD, Australia; ^3^Department of Culture, Communication and Media, Institute of Education, University College London, London, United Kingdom

**Keywords:** early childhood education and care, music education, early childhood educators, music beliefs and value, music practices in early childhood education and care

## Abstract

This paper reports the findings of a study that aimed to identify the music beliefs and values of educators in early childhood education and care settings in Australia. The aims of the study were 2-fold: to adapt and pilot a survey of music beliefs and values which might be implemented subsequently nationally in childcare settings; and, secondly, to identify the music beliefs and values held by early childhood and care educators concerning music in children's learning. The research questions that guided this component of the study were: What is the profile of early childhood and care educators? What beliefs and values for music engagement are held by early childhood and care educators? What shapes early childhood and care educators' music beliefs and values? Findings indicated that educators' beliefs and values on all items are above the mid-point indicating overall positive attitudes toward music despite the majority having no formal qualifications in music or a history of instrumental performance and/or singing. Given the overall positive attitudes toward music we suggest there is enormous potential within this population for further professional learning and development targeted at music and its potential wider benefits in young children's learning and lives.

## Introduction

In recent years there has been a documented steady decline in the provision of music education in the pre-service training of early childhood educators in Australia (Letts, 2015). Teacher education programs in early childhood have seen a reduction from some 72 h of music education over a 4-year Bachelor degree in 1988 (University of Tasmania, as one example) to current figures ranging from 0 to 17 h in total (Letts, 2015). Within the early education and care sector, professional qualifications such as certificates and diplomas have little consideration of music as either a content area or a teaching and learning strategy (Letts, 2015). Paradoxically, there is a substantial and growing body of literature that evidences the contributions of music learning and engagement to young children's development across a range of factors (for example, Moreno et al., 2009; Brown et al., 2010; Brown and Sax, 2013; Williams et al., 2015; Bugos and DeMarie, 2017).

Concurrent with this documented decline in educator preparation to teach and use music in early childhood education and care, there has been increasing recognition of the role early education and care plays in producing positive long-term development and learning outcomes (cf. Siraj-Blatchford et al., 2011). Governments internationally (Schober and Stahl, 2014; Department for Education, 2015) have focused on measuring and promoting access to quality early education and care; a development largely driven by the OECD. Australia is no exception to this with the Council of Australian Governments agreeing in 2009 to a National Early Childhood Development Strategy entitled *Investing in the early years* (Council of Australian Governments, 2009). Developments over the subsequent years include a bi-partisan initiative from successive federal governments to implement an *Early Years Learning Framework* (Department of Education Employment and Workplace Relations, 2009), an Educators' guide to use this framework (Department of Education Employment Workplace Relations, 2010), and a national monitoring and reporting process that assesses all childcare providers in the country against set criteria and quality standards (Australian Children's Education and Care Quality Authority (ACECQA), 2016). In short, there is significant recognition of the importance of early years learning and engagement and a strong commitment from a range of agencies in Australia to pursue a national agenda for improvement.

Despite the aforementioned body of evidence attesting to the importance of music in early learning and life, there is little recognition in official early years policy on the developmental role of music in early learning and engagement. Furthermore, there remains a concomitant lack of education and training for contemporary educators in the early childhood sector on how they might use music effectively as an educational tool. As an initial step to address this challenge, this paper aims to identify the values and beliefs of early childhood educators concerning their use of music. This will allow for a clearer and deeper understanding of the factors that facilitate or constrain the use of music in early childhood education and care programming.

This paper arises from a national investigation of young Australian children's experiences and engagement with music in the home, in Music Early Learning Programs (MELPs), and childcare (Barrett and Welch, 2013–2016). The overall investigation seeks to identify the ways in which Australian children and their families engage with music in these diverse settings, whilst noting the outcomes of such experience and engagement and the role of music-making in family life and parenting. The current paper reports findings from one strand of this project. Specifically, the analysis of music provision in long day-care settings in metropolitan and regional Queensland. The study focused on the experience, qualifications, and music beliefs and values of educators in these settings.

## Theoretical Background

Internationally, UNESCO (2016) has highlighted four main profile areas by which early childhood development can be assessed. These are executive function, social and emotional development, motor development, and early literacy and numeracy. There is a growing database of research literature that demonstrates how each of these profile areas can be nurtured through sustained engagement in musical activity. This is evidenced in studies on children's executive function (Moreno et al., 2011; Zuk et al., 2014; Bowmer et al., 2018), social and emotional development (Hallam, 2010; Barrett, 2011, 2016, 2017; Welch et al., 2014), motor development (Derri et al., 2001) and early literacy and numeracy (Anvari et al., 2002; Moritz et al., 2013; Williams et al., 2015; Cohrdes et al., 2016).

In addition, it has been reported that early music experiences can have a beneficial impact on a wide range of developmental features embracing cognitive, emotional, physical, and social domains. Example studies include those by Bengtsson et al. (2005), Chen et al. (2012), Creech et al. (2016), Dingle et al. (2012), Eerola and Eerola (2013), Forgeard et al. (2008), Fujioka et al. (2006), Gaser and Schlaug (2003), Gordon et al. (2015), Habib et al. (2016), Halwani et al. (2011), Hetland (2000), Ho et al. (2003), Hyde et al. (2009), Knight et al. (2016), Masataka and Perlovsky (2012), Moreno and Besson (2006), Moreno et al. (2009), Nutley et al. (2014), Osborne et al. (2016), Pantev et al. (2001), Paulson et al. (2013), Rickard et al. (2010), Roden et al. (2012), Saunders et al. (2014), Schlaug et al. (2005), Seinfeld et al. (2013), Tierney et al. (2013), Trappe (2012), Welch et al. (2014), Welch et al. (2015), Wetter et al. (2009), and Williams et al. (2015). For overviews of such impacts, see Hallam (2015), Schlaug (2015), Silvia et al. (2016) and—for a more discursive narrative—see Henriksson-Macauley (2014).

Furthermore, such benefits have been documented in studies that have controlled for socio-economic status (SES) and ethnicity. Key features in such studies are that beneficial music making is characterized by being sustained, active (singing and playing instruments), involves generative opportunities (composing and improvising) whether undertaken in groups or individually, and is a “fun” positive experience for children (cf. Barrett, 2012; Hallam, 2015). Additionally, recent research indicates that individual and shared music making in family settings contributes to positive parenting practices (Barrett, 2009), and early identity development in young children (Barrett, 2011, 2016, 2017). For example, a large-scale Australian study suggested that children who participate in shared music making at age three are better prepared for school-related experiences at age five (Williams et al., 2015). Overall, the body of research indicates that music is commonly a key component in young children's learning and development and can be a vital tool in the learning and care practices of early childhood educators. Collectively, these studies (and others) are building an evidence base of music's potential and actual impacts on different aspects of aural perception related to sound and language, verbal memory, spatial reasoning, self-regulation, pro-social skills, and aspects of general school-related attainment.

Paradoxically, other literature (cf. Letts, 2015) indicates that music education is barely addressed in the pre-service preparation of early childhood educators in University and Technical and Further Education (TAFE) settings, both in Australia and (often) internationally. Research suggests that teachers of young children in community and school settings often have limited experience of music education, other than being able to draw on their own personal experience. Such experience, however, has its limitations, often leading to a reported “lack of confidence” in generalist music educators who are working in early childhood education and care (ECEC) settings and primary schools (Mills, 1989; Hennessy, 2000; McCullough, 2006; Seddon and Biasutti, 2008; Stakelum, 2008; Hallam et al., 2009; Stunell, 2010; Welch and Henley, 2014). Furthermore, this reported “lack of confidence” in the teaching of music tends not to be addressed sufficiently in Primary teachers' initial teacher education (pre-service) courses (e.g., Ballantyne and Packer, 2004; Ballantyne, 2006).

Teachers' beliefs concerning the nature of child education, development, and teaching and learning, are powerful shaping forces in their classroom practices. Early childhood pre-service teachers' beliefs about music as an active developmental tool rather than mere enrichment are shaped by their experience and knowledge of music (Austin and Reinhardt, 1999; Kim and Kemple, 2011). Investigation into 21 early childhood educators' self-efficacy beliefs for teaching across different subject areas indicated significantly lower scores for the arts in comparison to their perceived confidence for teaching mathematics and English (Garvis and Pendergast, 2011). Overall however, little is known of the music beliefs of early childhood education and care (ECEC) practitioners, particularly those working with children prior to entry into formal schooling. This investigation seeks to address this gap.

## Aims

The research aims for this study were 2-fold: first, to adapt and pilot a survey of music beliefs and values which might be implemented subsequently nationally in childcare settings; and, secondly, to identify the music beliefs and values held by early childhood and care educators concerning music in children's learning. The research questions that guided this component of the study were:

1. What is the profile of early childhood and care educators?
2. What beliefs and values for music engagement are held by early childhood and care educators?
3. What shapes early childhood and care educators' music beliefs and values?

## Method

### Participants

Data collection took place at seven, long (i.e., extended) day-care centers that were located across metropolitan and regional Queensland. A total of 88 participants (87 females and one male) each completed two questionnaires with the assistance of a project researcher. Purposive stratified sampling was conducted via deliberate selection of educators from rural and urban childcare center locations in proportion to the number of rural and urban childcare centers operating in Queensland. This was implemented with the intention of capturing a diverse cross-section of socioeconomic status, early childhood music education philosophies and business models (including not-for-profit, commercial, and community models). Center Directors^1^ (*n* = 10), lead educators (*n* = 32), and general educators (*n* = 46) constituted the final study participants.

### Measures

Two questionnaires were completed. The first was a demographic survey, which asked for information about practitioner educational qualifications, current (if any) education being undertaken, employment status, years of experience in the early childhood education sector, and past and present music engagement. Following this, participants completed the Music Beliefs Questionnaire (MBQ). The MBQ is a 37-item survey, which gauges beliefs and values concerning the role of music within early childhood. Modified from the Austin and Reinhardt (1999) scale, the 37 items were designed to measure three key construct areas. These pertained to the beneficial outcomes of music for children in (1) creative and cultural development, (2) quality of life, and (3) social and emotional development. Responses to each statement were indicated on a 7-point Likert-type scale (1 = *very untrue of what I believe*; 7 = *very true of what I believe*; see Appendix A).

The MBQ piloted in the present study was an adaptation of Austin and Reinhardt's (1999) scale, originally designed to measure the music philosophy beliefs of pre-service teachers. A number of items in the Austin and Reinhardt scale were modified to suit the current study population and context, with an additional question included concerning music and special education. Items sought to gauge participants' beliefs about music by investigating concepts regarding: the outcomes of music education, music education significance for childhood development, music education relevance for child psychology, and music education importance in the creation and promotion of pro-social skills. Adaptations made for the revised scale also aimed to capture changes inherent in early childhood education theory, practice and policy that have occurred over time and across different countries of implementation. For example, Question 21 “*Music education supports the development of a child's identity”* reflects recent research that focuses on the formative role of music in identity (Hargreaves et al., 2017) and specifically in young children's identity (Barrett, 2011, 2016, 2017). Similarly, Question 36 “*Music education offers a way to include children with special learning needs”* acknowledges current policy and practice concerning inclusiveness in classrooms (Devarakonda, 2012; Jellison, 2018).

### Analytic Approach

This study focused on illuminating educators' (a) demographic profile, (b) music engagement beliefs and values held, and (c) factors shaping music beliefs and values. Analyses were conducted in three initial stages. The first provided descriptive statistics on demographic details that outlined the profile of early childhood and care educators from the study (see Table 1). Exploration was also performed of the basic descriptive statistics from individual MBQ items in order to identify educators' highest and lowest endorsed beliefs regarding music (see Table 2). This provided general insight into the perceived outcomes and values attached to music education by these Australian early childhood and care educators.

**Table 1:** Demographic profile of early childhood and care educators: frequency (*n*) and percentage (%) of key categorical characteristics in the analytic sample.

**Characteristic**	***n***	**%**
**Identified Gender**		
Female	87	98.86
Male	1	1.14
**Age of Participant**		
18–24	15	17.05
25–34	25	28.41
35–44	19	21.59
45–54	16	18.18
55–64	10	11.36
65+	2	2.27
Did Not State	1	1.14
**Educator Role**		
Director	10	11.36
Lead Educator	32	36.36
General Educator	46	52.27
**Educator Years of Experience**		
0–4 years	24	27.27
5–9 years	26	29.55
10–14 years	20	22.73
15–19 years	9	10.23
20+ years	9	10.23
**Employment Status**		
Full-Time	56	63.64
Part-Time	24	27.27
Casual	8	9.09
**Child Care Site Location**		
Urban	65	73.86
Rural	23	26.14
**Highest Qualification Level Achieved**		
Certificate/Diploma	69	78.41
Bachelors Degree	14	15.91
Postgraduate Degree	4	4.55
Missing	1	1.14
**Educator Qualification Field of Study**		
Early Childhood Education	36	40.91
Child Care	27	30.68
Primary/Secondary Education	7	7.95
Nursing	1	1.14
Other	9	10.23
None/not complete	8	9.09
**Educator Current Study Status**		
Currently Studying	18	20.45
Not Currently Studying	70	79.55
**Educator Current Field of Study**		
Early Childhood Education	11	12.50
Child Care	4	4.55
Primary/secondary Education	1	1.14
Other	2	2.27
Not Currently Studying	70	79.55
**Formal Music Qualifications**		
Yes	2	2.27
No	86	97.73
**Ever Learnt Instrument/Sung**		
Yes	42	47.73
No	46	52.27
**Currently Play Musical Instrument/Sing**		
Yes	14	15.91
No	28	31.82
Never Played/Sung	46	52.27

*N = 88. Percentages may not sum to 100.00% across categories within a demographic variable due to rounding*.

**Table 2:** Descriptive statistics for highest- and lowest-rated items by early childhood and care educators on the music beliefs questionnaire (MBQ).

**Scale item**	**Mean (*SD*)**	**Minimum**	**Maximum**
**HIGHEST-RATED MUSIC BELIEFS**			
Music education allows children to have fun (Q27)	6.74 (0.47)	5.00	7.00
Music education enables children to develop their musical ability (Q14)	6.50 (0.66)	4.00	7.00
Music education offers a way to include children from diverse cultures (Q3)	6.49 (0.68)	4.00	7.00
Music education provides children with opportunities to improve their self-esteem (Q2)	6.48 (0.66)	4.00	7.00
Music education encourages children to be creative (Q16)	6.47 (0.68)	4.00	7.00
Music education helps to develop children's self-confidence (Q10)	6.45 (0.71)	4.00	7.00
Music education provides children with a means of self-expression (Q15)	6.45 (0.71)	4.00	7.00
Music education is an important part of a holistic approach to education (Q28)	6.39 (0.67)	4.00	7.00
Music education encourages children to use their imagination (Q31)	6.39 (0.70)	4.00	7.00
Music education offers a way to include children who sometimes have trouble playing in a group with other children (Q37)	6.38 (0.76)	4.00	7.00
**LOWEST-RATED MUSIC BELIEFS**			
Music education enables children to make meaning of their experiences of the world (Q7)	5.72 (1.01)	3.00	7.00
Music education supports children to learn to control their behavior (Q4)	5.72 (1.04)	4.00	7.00
Music education helps children to persist with challenging tasks (Q30)	5.76 (1.01)	4.00	7.00
Music education helps children develop problem-solving skills (Q29)	5.78 (1.03)	3.00	7.00
Music education enables children to improve the quality of their lives (Q19)	5.82 (1.03)	4.00	7.00
Music education encourages children's understanding of different symbol systems (Q5)	5.90 (0.97)	4.00	7.00
Music education enables children to understand more sophisticated and complex music (Q33)	5.95 (0.93)	3.00	7.00
Music education supports children's skills in managing their own emotions (Q35)	5.99 (0.94)	4.00	7.00
Music education is valuable in itself and needs no other justification (Q18)	6.00 (1.08)	1.00	7.00
Music education supports the development of a child's identity (Q21)	6.01 (0.82)	4.00	7.00

*N = 88. All items on the MBQ were scaled from 1 (very untrue of what I believe) to 7 (very true of what I believe)*.

**Figure 1:**
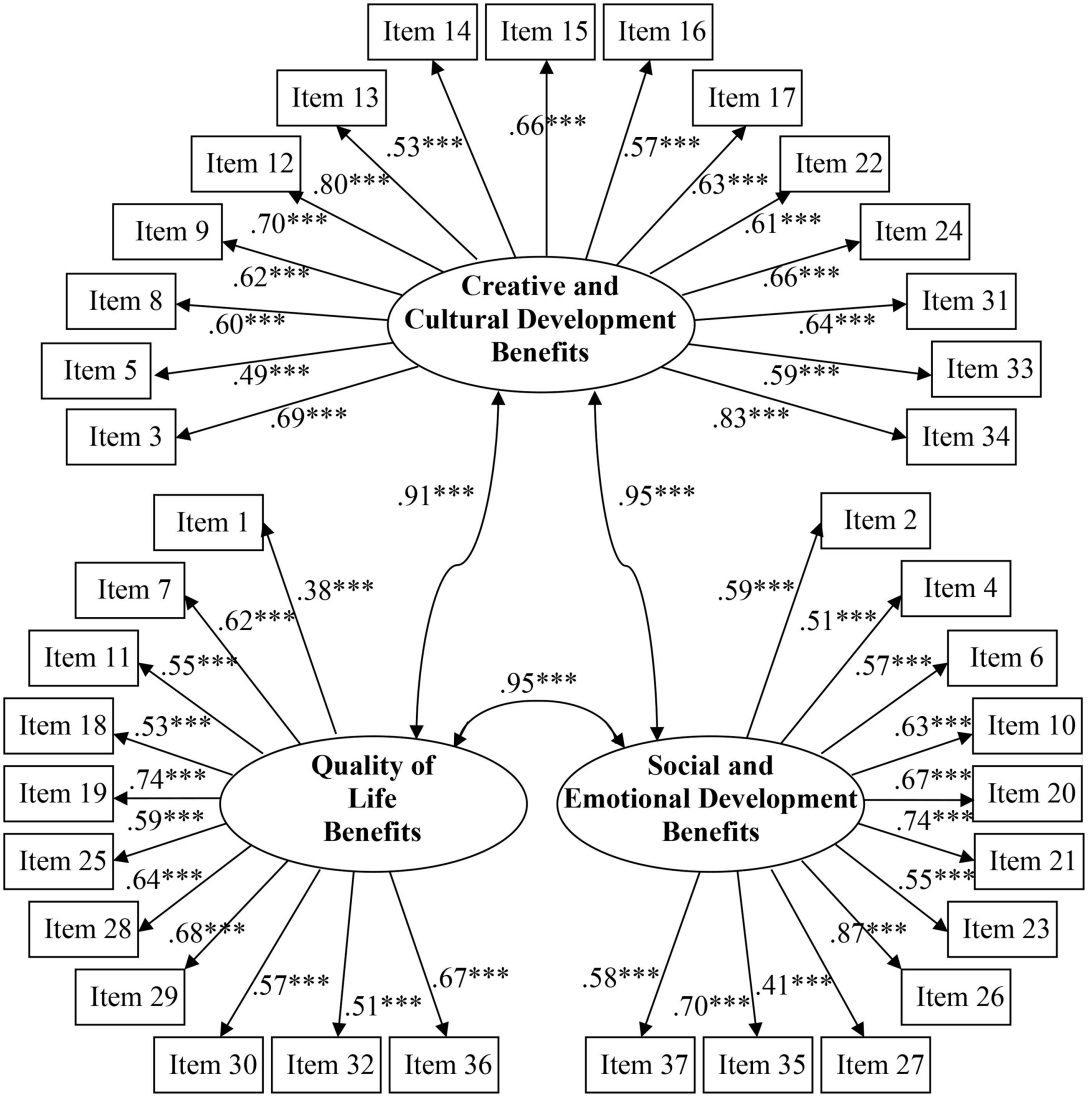
Confirmatory factor analysis of the 37 Music Belief Questionnaire items: The hypothesized three-factor solution. All given regression weights are standardized parameter estimates. ***p < 0.001.

In the second stage, a confirmatory factor analysis (CFA) was performed to validate the intended three-factor structure for the 37 MBQ survey items (see Figure 1 and **Table 4**). This model was compared and contrasted against an alternative single-factor structure. The single-factor model was found to provide an equally plausible explanation for the observed data, with both models displaying adequate overall model fit. However, the three-factor model demonstrated very high intercorrelations amongst its proposed latent constructs, almost to the point of singularity. Therefore, the more parsimonious single-factor structure was adopted. Given that some of the fit indices from both models were less than desirable, an exploratory factor analysis (EFA) using principal axis factoring extraction and direct oblimin (oblique) rotation was also conducted. This allowed for a data-driven approach to determine the underlying factor structure. Results provided by the scree plot further corroborated the use of a single-factor model to best represent the data. Thus, a unified MBQ scale score was utilized in further analyses.

The third stage of analysis made use of this overall MBQ score as the key outcome variable in a series of linear regressions, to explore which factors predict the general music beliefs and values held by early childhood and care educators regarding the benefits of music for young children (see **Table 6**). Specifically, the MBQ scale score was regressed onto educator age groupings (18–24, 25–34, 35–44, 45–54, 55–64, 65+ years), educator role (higher level educator [directors and lead educators], general educator), educator years of experience (0–4, 5–9, 10–14, 15–19, 20+ years), whether the educator had ever learnt a musical instrument or sung in choir (no, yes), highest level post-school qualification achieved by the educator (certificate/diploma, bachelor's degree, postgraduate degree), and the regional site from which the educator worked (rural, urban). These analyses were performed both as separate bivariate regressions with the individual predictors and as a standard multiple regression with all predictors entered simultaneously. This exploratory analytic approach allowed us to investigate not only the individual contributions from each factor, but also to identify any redundancy among predictors. As such, it permitted illumination of the key facilitating factors promoting music beliefs and values of these early childhood and care educators and, thereby, provided an important contribution to our current understanding of the area. In the next section, we present the study results and discussion, linking findings back to the core research questions.

## Results

### What Is the Demographic Profile of Early Childhood and Care Educators?

Descriptive statistics for the demographic profile of the analytic sample are presented in Table 1. The overwhelming majority of educator participants were female, which reflects the current over-representation of females globally in the early childhood education and care sector (Peeters et al., 2015). While slightly more participants were aged between 25 and 34 years, the range did vary. Indeed, the four youngest categories spanning 18–54 years best captured participants' age profile. Educators' years of experience also varied, but were best represented by the three lowest options spanning 0–14 years' experience. An additional basic descriptive analysis on the continuous variable of length of current employment revealed a more nuanced view on this. It showed that the average time educators had worked at their current care center was 6.25 years (*SD* = 4.81).

The majority of early childhood and care educators were employed on a full-time basis, with far fewer employed part-time or casually. Employment for educators in urban child care centers was far more common than in rural sites, which likely reflected service demand. Educators most frequently achieved a certificate or diploma as their highest qualification, with relatively few achieving a bachelor's or postgraduate university degree. Of those who had completed study beyond high school, the vast majority had chosen fields relevant to their current career, such as early childhood education, child care and Primary/Secondary education. A minority of educators were presently studying while concurrently working, either obtaining their ECEC qualifications or completing higher degrees usually with a clear focus on the relevant study fields of early childhood education, child care, and Primary/Secondary education.

In relation to music knowledge, few educators reported having attained any formal qualifications in music. However, approximately half of all participants stated some personal experience with music education, either having learnt to play an instrument and/or having sung in a choir themselves. Further basic descriptive analyses revealed that the average length of time that any had learnt a musical instrument or sung in choir was 3.42 years (*SD* = 2.66). Of this group, only one-third reported that they currently played and/or sang. This suggested that while half of participants had past experience with music, two-thirds of these had not continued active musical engagement. With this demographic profile in mind, we next sought to understand the beliefs and values these educators held about music education in the lives of young children in an effort to comprehend their teaching motivations and ethos better.

### What Beliefs and Values Regarding Music Engagement Are Held by Early Childhood and Care Educators?

To evaluate the beliefs and values held by these educators pertaining to the role of music education in young children's early learning and development, a 37-item Music Beliefs Questionnaire (MBQ) was administered to study participants. It was observed that on the 1–7 response scale, the majority of items (78.38%) displayed mean scores of 6.00 (*true of what I believe*) or higher, with the lowest mean of 5.72 being shown for two items (i.e., “*Music education supports children to learn to control their behavior*” and “*Music education enables children to make meaning of their experiences of the world*”). These consistently high mean item values, coupled with low standard deviations, suggested that the educators in this analytic sample expressed extremely positive, shared, global beliefs, and values regarding music and its place in the education of young Australian children. To help shed light on the most and least firmly held beliefs within these generally very high values, Table 2 presents the 10 highest and 10 lowest endorsed item statements from the MBQ.

As seen from these results, most strongly endorsed beliefs by early childhood and care educators included the concept of music as: (a) an essential creative outlet for young children; (b) a useful tool for the social inclusion of children; as well as (c) a method by which to bolster children's emotional development (i.e., self-esteem and self-confidence) in a fun and non-threatening space. Slightly less strongly endorsed were the utilitarian and adaptable skill aspects that music has to offer. Specifically, participants assigned relatively lower overall mean ratings to statements concerning ideas that children could use the skills gained from music education to understand the world, exert self-discipline (i.e., emotional and behavioral control), and bolster resilience, identity development, and quality of life. Furthermore, lower support was shown for concepts that key skills gained from music would transfer to aid general problem-solving, deciphering different symbol systems, or understanding more complex music (although it should be noted that these ratings still sat above the mid-point of the seven-point scale). Indeed, music education appeared not to be valued highly in and of itself, but rather viewed more as a fun and playful way to develop children's social and creative aspects to round out—rather than enhance transferable skills that can help develop and promote facets of—their traditional academic education.

This viewpoint appears to conflict somewhat with research findings that have reported the value of sustained music education in the nurturing of early literacy and numeracy (cf. Anvari et al., 2002; Moritz et al., 2013; Williams et al., 2015; Cohrdes et al., 2016), social and emotional development (Hallam, 2010; Welch et al., 2014), promotion of children's executive functioning (Moreno et al., 2011; Zuk et al., 2014; Bowmer et al., 2018), and motor skill development (Derri et al., 2001).

### Creation of the Music Beliefs Questionnaire (MBQ) Scale Score

In line with the notable ceiling effects observed above, preliminary assessment of the score distributions for each MBQ item revealed that a large number displayed issues regarding skewness, and kurtosis. Specifically, the item *z*-scores for skewness and kurtosis were tested following the recommendations of Tabachnick and Fidell (2013), where an absolute value exceeding 3.29 was deemed significant (i.e., representative of the *p* < 0.001 criterion). This revealed that 19 of the 37 MBQ scale items exhibited significant skew, whilst seven showed significant kurtosis (see Table 3). Given this violation of the assumption of criterion normality, a bootstrap approach was adopted to the ensuing confirmatory factor analysis (CFA) using 5,000 re-samples, employing the Bollen-Stine method, and robust bootstrap-adjusted fit indices recommended for use with non-normal data (Walker and Smith, 2017).

Table 3Descriptive statistics, item loadings for single-factor model, and Spearman's Rho Intercorrelations for the MBQ scale items (*N* = 88).

**Table:** 

**MBQ Item**	***M* (*SD*)**	**Skewness**	**Kurtosis**	**Item Loading**	**Item 1**	**Item 2**	**Item 3**	**Item 4**	**Item 5**	**Item 6**	**Item 7**	**Item 8**	**Item 9**
1.	6.38 (0.85)	−5.40^***^	2.71	0.38^**^	–								
2.	6.48 (0.66)	−4.43^***^	2.40	0.60^***^	0.45^***^	–							
3.	6.49 (0.68)	−4.68^***^	2.26	0.67^***^	0.34^**^	0.52^***^	–						
4.	5.72 (1.04)	−0.86	−2.22	0.52^***^	0.39^***^	0.33^**^	0.51^***^	–					
5.	5.90 (0.97)	−2.48	−0.95	0.47^***^	0.18	0.42^***^	0.46^***^	0.28^**^	–				
6.	6.09 (0.88)	−3.12	1.08	0.58^***^	0.33^**^	0.35^**^	0.34^**^	0.44^***^	0.38^***^	–			
7.	5.72 (1.01)	−1.72	−1.06	0.61^***^	0.27^*^	0.40^***^	0.41^***^	0.45^***^	0.36^**^	0.62^***^	–		
8.	6.26 (0.89)	−5.23^***^	3.55^***^	0.61^***^	0.25^*^	0.35^**^	0.51^***^	0.46^***^	0.36^**^	0.50^***^	0.53^***^	–	
9.	6.14 (0.92)	−3.86^***^	1.32	0.57^***^	0.33^**^	0.47^***^	0.51^***^	0.34^**^	0.61^***^	0.30^**^	0.38^***^	0.50^***^	–
10.	6.45 (0.71)	−5.12^***^	3.51^***^	0.62^***^	0.49^***^	0.69^***^	0.47^***^	0.36^**^	0.35^**^	0.31^**^	0.43^***^	0.35^**^	0.41^***^
11.	6.20 (0.98)	−6.26^***^	6.41^***^	0.51^***^	0.59^***^	0.41^***^	0.45^***^	0.35^**^	0.24^*^	0.55^***^	0.43^***^	0.48^***^	0.46^***^
12.	6.24 (0.91)	−6.67^***^	9.21^***^	0.67^***^	0.46^***^	0.48^***^	0.64^***^	0.43^***^	0.33^**^	0.47^***^	0.37^***^	0.51^***^	0.47^***^
13.	6.13 (0.80)	−2.51	−0.08	0.78^***^	0.39^***^	0.44^***^	0.55^***^	0.51^***^	0.49^***^	0.50^***^	0.50^***^	0.56^***^	0.52^***^
14.	6.50 (0.66)	−4.75^***^	2.72	0.49^***^	0.35^**^	0.34^**^	0.40^***^	0.23^*^	0.40^***^	0.35^**^	0.40^***^	0.52^***^	0.50^***^
15.	6.45 (0.71)	−4.35^***^	1.32	0.63^***^	0.34^**^	0.46^***^	0.43^***^	0.28^**^	0.36^**^	0.40^***^	0.41^***^	0.60^***^	0.48^***^
16.	6.47 (0.68)	−4.37^***^	1.97	0.53^***^	0.31^**^	0.31^**^	0.32^**^	0.18	0.34^**^	0.29^**^	0.31^**^	0.45^***^	0.55^***^
17.	6.17 (0.83)	−3.19	0.31	0.62^***^	0.20	0.43^***^	0.49^***^	0.30^**^	0.39^***^	0.30^**^	0.44^***^	0.50^***^	0.47^***^
18.	6.00 (1.08)	−5.84^***^	7.75^***^	0.52^***^	0.36^**^	0.29^**^	0.34^**^	0.28^**^	0.36^**^	0.21^*^	0.22^*^	0.21	0.45^***^
19.	5.82 (1.03)	−1.02	−2.31	0.69^***^	0.31^**^	0.39^***^	0.48^***^	0.55^***^	0.39^***^	0.49^***^	0.53^***^	0.49^***^	0.39^***^
20.	6.13 (0.79)	−2.58	0.24	0.65^***^	0.25^*^	0.37^***^	0.27^*^	0.41^***^	0.30^**^	0.47^***^	0.54^***^	0.57^***^	0.30^**^
21.	6.01 (0.82)	−1.56	−1.06	0.73^***^	0.15	0.40^***^	0.38^***^	0.33^**^	0.50^***^	0.43^***^	0.47^***^	0.50^***^	0.34^**^
22.	6.15 (0.94)	−6.44^***^	8.53^***^	0.61^***^	0.33^**^	0.43^***^	0.47^***^	0.28^**^	0.33^**^	0.32^**^	0.36^**^	0.50^***^	0.42^***^
23.	6.22 (0.79)	−3.24	0.59	0.53^***^	0.24^*^	0.44^***^	0.29^**^	0.34^**^	0.21^*^	0.26^*^	0.23^*^	0.36^**^	0.22^*^
24.	6.15 (0.92)	−5.45^***^	7.06^***^	0.68^***^	0.37^***^	0.48^***^	0.48^***^	0.41^***^	0.40^***^	0.41^***^	0.43^***^	0.52^***^	0.50^***^
25.	6.30 (0.73)	−2.75	−0.24	0.57^***^	0.24^*^	0.34^**^	0.35^**^	0.34^**^	0.24^*^	0.43^***^	0.38^***^	0.39^***^	0.18
26.	6.16 (0.87)	−2.91	−0.49	0.86^***^	0.29^**^	0.50^***^	0.59^***^	0.39^***^	0.42^***^	0.45^***^	0.53^***^	0.66^***^	0.45^***^
27.	6.74 (0.47)	−5.63^***^	1.87	0.40^***^	0.25^*^	0.24^*^	0.21^*^	−0.01	0.18	0.31^**^	0.19	0.30^**^	0.21^*^
28.	6.39 (0.67)	−3.39^***^	1.35	0.62^***^	0.23^*^	0.32^**^	0.49^***^	0.21^*^	0.32^**^	0.40^***^	0.36^**^	0.57^***^	0.47^***^
29.	5.78 (1.03)	−1.99	−1.15	0.65^***^	0.24^*^	0.37^***^	0.36^**^	0.48^***^	0.46^***^	0.51^***^	0.46^***^	0.54^***^	0.42^***^
30.	5.76 (1.01)	−1.03	−2.02	0.55^***^	0.15	0.44^***^	0.35^**^	0.39^***^	0.41^***^	0.44^***^	0.40^***^	0.34^**^	0.41^***^
31.	6.39 (0.70)	−3.54^***^	0.78	0.63^***^	0.36^**^	0.46^***^	0.37^***^	0.24^*^	0.32^**^	0.41^***^	0.43^***^	0.55^***^	0.49^***^
32.	6.10 (0.95)	−3.72^***^	0.94	0.49^***^	0.18	0.10	0.19	0.38^***^	0.06	0.38^***^	0.41^***^	0.33^**^	0.23^*^
33.	5.95 (0.93)	−2.34	−0.13	0.60^***^	0.27^*^	0.35^**^	0.51^***^	0.42^***^	0.38^***^	0.47^***^	0.39^***^	0.37^***^	0.30^**^
34.	6.11 (0.84)	−2.26	−0.84	0.84^***^	0.39^***^	0.52^***^	0.61^***^	0.45^***^	0.49^***^	0.52^***^	0.61^***^	0.58^***^	0.46^***^
35.	5.99 (0.94)	−2.22	−1.19	0.69^***^	0.30^**^	0.32^**^	0.38^***^	0.35^**^	0.29^**^	0.48^***^	0.51^***^	0.48^***^	0.31^**^
36.	6.35 (0.71)	−2.47	−1.56	0.67^***^	0.39^***^	0.30^**^	0.51^***^	0.25^*^	0.28^**^	0.45^***^	0.30^**^	0.55^***^	0.38^***^
37.	6.38 (0.76)	−4.19^***^	1.29	0.56^***^	0.23^*^	0.30^**^	0.46^***^	0.19	0.17	0.31^**^	0.30^**^	0.47^***^	0.26^*^

**Table:** 

**MBQ Item**	**Item 10**	**Item 11**	**Item 12**	**Item 13**	**Item 14**	**Item 15**	**Item 16**	**Item 17**	**Item 18**	**Item 19**	**Item 20**	**Item 21**	**Item 22**	**Item 23**
1.														
2.														
3.														
4.														
5.														
6.														
7.														
8.														
9.														
10.	–													
11.	0.40^***^	–												
12.	0.48^***^	0.61^***^	–											
13.	0.52^***^	0.44^***^	0.69^***^	–										
14.	0.43^***^	0.24^*^	0.38^***^	0.54^***^	–									
15.	0.53^***^	0.39^***^	0.47^***^	0.57^***^	0.63^***^	–								
16.	0.37^***^	0.36^**^	0.37^***^	0.55^***^	0.63^***^	0.74^***^	–							
17.	0.52^***^	0.28^**^	0.39^***^	0.49^***^	0.50^***^	0.59^***^	0.55^***^	–						
18.	0.45^***^	0.38^***^	0.37^***^	0.43^***^	0.34^**^	0.42^***^	0.40^***^	0.40^***^	–					
19.	0.41^***^	0.47^***^	0.41^***^	0.48^***^	0.28^**^	0.31^**^	0.21	0.38^***^	0.49^***^	–				
20.	0.45^***^	0.39^***^	0.35^**^	0.43^***^	0.37^***^	0.53^***^	0.43^***^	0.52^***^	0.43^***^	0.68^***^	–			
21.	0.50^***^	0.24^*^	0.39^***^	0.53^***^	0.48^***^	0.51^***^	0.44^***^	0.59^***^	0.39^***^	0.53^***^	0.66^***^	–		
22.	0.41^***^	0.35^**^	0.50^***^	0.51^***^	0.46^***^	0.43^***^	0.43^***^	0.43^***^	0.44^***^	0.49^***^	0.50^***^	0.55^***^	–	
23.	0.39^***^	0.20	0.39^***^	0.44^***^	0.19	0.33^**^	0.19	0.38^***^	0.35^**^	0.46^***^	0.52^***^	0.43^***^	0.53^***^	–
24.	0.46^***^	0.39^***^	0.48^***^	0.64^***^	0.43^***^	0.47^***^	0.38^***^	0.52^***^	0.63^***^	0.65^***^	0.53^***^	0.48^***^	0.61^***^	0.59^***^
25.	0.45^***^	0.35^**^	0.37^***^	0.36^***^	0.34^**^	0.29^**^	0.25^*^	0.35^**^	0.37^***^	0.38^***^	0.43^***^	0.59^***^	0.38^***^	0.50^***^
26.	0.56^***^	0.44^***^	0.59^***^	0.71^***^	0.46^***^	0.56^***^	0.51^***^	0.63^***^	0.47^***^	0.53^***^	0.63^***^	0.69^***^	0.53^***^	0.55^***^
27.	0.35^**^	0.33^**^	0.41^***^	0.32^**^	0.44^***^	0.48^***^	0.47^***^	0.24^*^	0.28^**^	0.11	0.33^**^	0.25^*^	0.33^**^	0.28^**^
28.	0.36^**^	0.42^***^	0.49^***^	0.38^***^	0.35^**^	0.54^***^	0.47^***^	0.42^***^	0.54^***^	0.51^***^	0.64^***^	0.51^***^	0.57^***^	0.40^***^
29.	0.35^**^	0.40^***^	0.44^***^	0.53^***^	0.27^*^	0.35^**^	0.34^**^	0.40^***^	0.24^*^	0.56^***^	0.48^***^	0.57^***^	0.38^***^	0.42^***^
30.	0.36^**^	0.29^**^	0.47^***^	0.39^***^	0.22^*^	0.29^**^	0.31^**^	0.40^***^	0.16	0.45^***^	0.37^***^	0.48^***^	0.38^***^	0.48^***^
31.	0.46^***^	0.52^***^	0.55^***^	0.59^***^	0.37^***^	0.56^***^	0.52^***^	0.41^***^	0.23^*^	0.27^*^	0.49^***^	0.46^***^	0.43^***^	0.45^***^
32.	0.25^*^	0.38^***^	0.39^***^	0.40^***^	0.19	0.34^**^	0.31^**^	0.22^*^	0.24^*^	0.32^**^	0.38^***^	0.36^**^	0.24^*^	0.35^**^
33.	0.32^**^	0.40^***^	0.58^***^	0.56^***^	0.33^**^	0.36^***^	0.33^**^	0.44^***^	0.42^***^	0.54^***^	0.50^***^	0.52^***^	0.65^***^	0.50^***^
34.	0.53^***^	0.52^***^	0.57^***^	0.60^***^	0.48^***^	0.63^***^	0.49^***^	0.49^***^	0.51^***^	0.56^***^	0.60^***^	0.71^***^	0.54^***^	0.40^***^
35.	0.47^***^	0.35^**^	0.48^***^	0.58^***^	0.47^***^	0.40^***^	0.44^***^	0.38^***^	0.26^*^	0.47^***^	0.47^***^	0.60^***^	0.55^***^	0.40^***^
36.	0.35^**^	0.58^***^	0.51^***^	0.53^***^	0.40^***^	0.47^***^	0.42^***^	0.32^**^	0.50^***^	0.41^***^	0.41^***^	0.47^***^	0.48^***^	0.36^**^
37.	0.43^***^	0.47^***^	0.37^***^	0.34^**^	0.36^**^	0.42^***^	0.33^**^	0.46^***^	0.50^***^	0.39^***^	0.50^***^	0.49^***^	0.44^***^	0.46^***^

**Table:** 

**MBQ Item**	**Item 24**	**Item 25**	**Item 26**	**Item 27**	**Item 28**	**Item 29**	**Item 30**	**Item 31**	**Item 32**	**Item 33**	**Item 34**	**Item 35**	**Item 36**
1.													
2.													
3.													
4.													
5.													
6.													
7.													
8.													
9.													
10.													
11.													
12.													
13.													
14.													
15.													
16.													
17.													
18.													
19.													
20.													
21.													
22.													
23.													
24.	–												
25.	0.45^***^	–											
26.	0.62^***^	0.62^***^	–										
27.	0.22^*^	0.34^**^	0.44^***^	–									
28.	0.53^***^	0.40^***^	0.56^***^	0.39^***^	–								
29.	0.39^***^	0.43^***^	0.51^***^	0.13	0.41^***^	–							
30.	0.38^***^	0.34^**^	0.36^**^	0.11	0.33^**^	0.70^***^	–						
31.	0.42^***^	0.40^***^	0.68^***^	0.53^***^	0.38^***^	0.42^***^	0.36^**^	–					
32.	0.20	0.36^**^	0.46^***^	0.37^***^	0.36^***^	0.37^***^	0.30^**^	0.48^***^	–				
33.	0.55^***^	0.44^***^	0.57^***^	0.23^*^	0.51^***^	0.55^***^	0.53^***^	0.39^***^	0.40^***^	–			
34.	0.55^***^	0.59^***^	0.78^***^	0.37^***^	0.58^***^	0.53^***^	0.42^***^	0.55^***^	0.46^***^	0.54^***^	–		
35.	0.45^***^	0.48^***^	0.65^***^	0.31^**^	0.37^***^	0.49^***^	0.45^***^	0.54^***^	0.55^***^	0.52^***^	0.59^***^	–	
36.	0.48^***^	0.46^***^	0.60^***^	0.43^***^	0.66^***^	0.41^***^	0.25^*^	0.43^***^	0.41^***^	0.40^***^	0.65^***^	0.47^***^	–
37.	0.40^***^	0.54^***^	0.65^***^	0.42^***^	0.64^***^	0.28^**^	0.21	0.36^**^	0.41^***^	0.38^***^	0.59^***^	0.48^***^	0.75^***^

† p < 0.10

* p < 0.05

** p < 0.01

*** *p < 0.001*.

In an attempt to confirm the intended three-factor structure of the MBQ, the questionnaire items were submitted to a CFA. The proposed model was evaluated for fit regarding the 37 items measuring the three latent constructs they were designed to assess; namely, participant educators' beliefs and values in relation to the benefits engendered by music on children's (1) creative and cultural development, (2) quality of life, and (3) social and emotional development. These three factors aligned closely with—and were adapted from—those of Austin and Reinhardt (1999), who proposed the three factors of pre-service teachers' music philosophical beliefs to be (1) aesthetic benefits, (2) quality-of-life benefits, and (3) social-emotional benefits, respectively. Therefore, the previous administration of the Austin and Reinhardt scale from which our new measure was adapted formed the *a priori* theoretical basis that guided our choice of (a) number of factors, and (b) which items loaded onto each factor. As such, 15 items were specified to load on the creative and cultural development benefits factor (α = 0.91, for items and content see Table 4), 11 items were confined to load on the quality of life benefits factor (α = 0.85), and the remaining 11 items were constrained to the social and emotional development benefits factor (α = 0.87). Each scale item was specified as loading on only one factor and, given that all latent constructs formed underlying facets regarding beliefs about music, the three factors were permitted to correlate. Intercorrelations amongst all the scale items are presented in Table 3, where these were performed as Spearman rho associations due to the non-normal nature of many item response distributions.

**Table 4:** Proposed three factors and associated MBQ items submitted to confirmatory factor analysis (CFA).

**FACTOR 1: CREATIVE AND CULTURAL DEVELOPMENT BENEFITS**
Music education offers a way to include children from diverse cultures (Q3)
Music education encourages children's understanding of different symbol systems (Q5)
Music education supports children's use of alternative forms of communication (Q8)
Music education enhances children's awareness and understanding of the arts (Q9)
Music education increases children's awareness of other cultures (Q12)
Music education provides children with new ideas and skills that can be used in their play (Q13)
Music education enables children to develop their musical ability (Q14)
Music education provides children with a means of self-expression (Q15)
Music education encourages children to be creative (Q16)
Music education encourages children to participate in home and community music making (Q17)
Music education helps children to appreciate and understand the role of music in their culture (Q22)
Music education increases the satisfaction that children are able to derive from music (Q24)
Music education encourages children to use their imagination (Q31)
Music education enables children to understand more sophisticated and complex music (Q33)
Music education provides children with access to a different form of intelligence or way of knowing (Q34)
**FACTOR 2: QUALITY OF LIFE BENEFITS**
Music education helps children develop and improve their motor-coordination skills (Q1)
Music education enables children to make meaning of their experiences of the world (Q7)
Music education helps children learn in other content areas (e.g., early literacy, numeracy) (Q11)
Music education is valuable in itself and needs no other justification (Q18)
Music education enables children to improve the quality of their lives (Q19)
Music education helps develop children's ability to focus their attention (Q25)
Music education is an important part of a holistic approach to education (Q28)
Music education helps children develop problem-solving skills (Q29)
Music education helps children to persist with challenging tasks (Q30)
Music education enhances the physical well-being of children (Q32)
Music education offers a way to include children with special learning needs (Q36)
**FACTOR 3: SOCIAL AND EMOTIONAL DEVELOPMENT BENEFITS**
Music education provides children with opportunities to improve their self-esteem (Q2)
Music education supports children to learn to control their behavior (Q4)
Music education helps children to learn about and understand emotions (Q6)
Music education helps to develop children's self-confidence (Q10)
Music education helps children develop relationships with others (Q20)
Music education supports the development of a child's identity (Q21)
Music education teaches children how to work together as a team (Q23)
Music education helps children to develop social skills (Q26)
Music education allows children to have fun (Q27)
Music education supports children's skills in managing their own emotions (Q35)
Music education offers a way to include children who sometimes have trouble playing in a group with other children (Q37)

The latent structure underlying the music belief and value items was evaluated using a three-factor CFA model. This hypothesized structure provided good fit, whereby the observed data did not differ significantly from the proposed model, χ^2^(626, *N* = 88) = 1,274.80, *p* = 0.169. However, the remaining fit indices did not meet the recommended 0.95 threshold for acceptable fit (Hu and Bentler, 1999): CFI_adj_ = 0.707, IFI_adj_ = 0.712, TLI_adj_ = 0.688. Furthermore, the residual index for the proposed model was not below the recommended 0.06 cut-off (Hu and Bentler, 1999): RMSEA_adj_ = 0.109.

Examination of the standardized parameter estimates showed that all of the items loaded significantly onto their respective hypothesized factors yet ranged somewhat in magnitude from 0.38 to 0.87 (see Figure 1). More specifically, only six items displayed strong loadings on the intended factor (i.e., >0.70), with three items demonstrating a weak loading (i.e., >0.30) and the remaining 28 showing moderate loadings on the prescribed factor (i.e., >0.50). In addition, while the three constructs were predicted to be related, these latent factors were too highly correlated with one another, almost to the point of singularity. These combined results suggested that, rather than the proposed three-factor solution, the MBQ items may be better captured by a single-factor scale, where the three factors are collapsed into one unifying latent construct assessing general educator beliefs and values held in relation to music. As such, this was evaluated as an alternative factor structure and compared to the original three-factor model.

To evaluate if a single general music beliefs factor better accounted for the current data, a one-factor solution was tested. For this, all 37 MBQ items were specified to load upon the one factor (α = 0.95). Similar to the three-factor structure, despite the overall model fitting the data well, χ^2^(629, *N* = 88) = 1,277.87, *p* = 0.155, the other indices did not meet the recommended thresholds for good fit: CFI_adj_ = 0.707, IFI_adj_ = 0.712, TLI_adj_ = 0.690, RMSEA_adj_ = 0.109. As can be seen in Table 3, like the three-factor model, standardized item loadings for the single-factor model were all significant, but ranged in magnitude from 0.38 to 0.86. In this, four items presented with strong loadings, 28 items demonstrated moderate loadings, and the final five items exhibited weak loadings. Based upon the content of the items loading upon this single factor, the underlying construct perceived as captured was general favorable educator beliefs and values regarding the role of music in early childhood. Therefore, this factor was assigned the label “general favorable music beliefs.”

The change in chi-square between the three-factor and one-factor models revealed no significant difference, Δχ^2^(3) = 3.06, *p* = 0.382. These findings showed there was no significant improvement in model fit by the more constrained and complex hypothesized three-factor model over the one-factor model, as both provided an equally plausible theoretical account for the data. Consequently, use of the three latent constructs of creative and cultural development benefits, quality of life benefits, and social and emotional development benefits, did not capture and explain the present data set better than a single unified general set of beliefs about the role of music in childhood development. Taken together, these results suggested that the more parsimonious single-factor structure was preferred over the three-factor model and, as such, scores on the MBQ were calculated by averaging across all the items to obtain an overall single general measure of favorable beliefs and values held regarding the role of music in early childhood^2^.

It should be noted that neither of the tested models provided an adequate fit in terms of the absolute, incremental and residual fit indices. Therefore, neither provided a truly comprehensive account of the current data. This problem may be reflective of two core issues. Firstly, the factors may not be clearly defined by each set of items, as indicated by the predominantly moderate item loadings. This could suggest that the items did not measure the underlying construct as well as intended. This would have caused a discrepancy between the proposed model and the observed data, resulting in the lower fit indices, and higher residual indices than the recommended threshold cut-offs that was seen.

Secondly, the general lack of score variability—as seen from the restriction of range on many scale items—may have interfered with the appropriate clustering of scores that serve to differentiate the various factors more cleanly. This may have obstructed identification of the true magnitude of correlations among the various scale items and, similarly, identification of the groups of intercorrelations that form the basis of factors. This could explain why the three-factor model failed to emerge as the superior explanation for the data, as the data itself may not have contained the necessary spread of scores to better determine and discriminate among more than one factor. This is especially likely given that all scores gathered were at the high end of the 1–7 response scale and thus would have produced high intercorrelations with one another, assuming each participant provided the same or very similar scores to all items in the MBQ scale, representative of their overall general favorable music beliefs. This in turn, could also explain the very high correlations observed between the three latent constructs within the proposed three-factor model.

It also should be acknowledged, however, that these formulations are merely conjecture based upon the presence of restricted range issues within the present data set. In cases where score variability is lacking, it is more prudent and thus we would argue best practice to adopt an overall scale measure that has been *shown* to capture the current data, rather than make use of separate proposed scale factors (i.e., subscales) that are only *theorized* to bear out if greater score variability had have been achieved, as the latter may not form valid subscales. Reference to other similar scales for guidance in this matter—although wise—may not prove useful, as there exists some constructs for which people have a general tendency to express highly positive attitudes. Therefore, scores reflecting such variables will always be restricted in range and negatively skewed in its natural form. As such, use of scales measuring these types of constructs will be accompanied by extreme difficulty when trying to achieve the full range of response score options from participants and, consequently, will impede the ability to tease apart and identify the underlying theoretical constructs—should more than one exist to explain the data. As stated above, the most conservative and safest method in such instances is use of a single general measure to represent participants' views. Therefore, this approach was adopted as we sought to explore the early childhood and care educator characteristics that informed their beliefs and values about music.

### What Shapes Early Childhood and Care Educators' Music Beliefs and Values?

To investigate which factors may influence educators' general beliefs and values about music, a series of six bivariate linear regressions were performed. Specifically, participants' average MBQ scores were regressed upon each of the predictors of (a) educator age, (b) educator role, (c) years of educator experience, (d) whether the educator had ever learnt a musical instrument or sung, (e) highest level of post-school qualification achieved by the educator, and (f) childcare regional site. These analyses were conducted individually to identify firstly any important contributing factors to the shaping of overall music beliefs.

Preliminary zero-order correlations among overall MBQ scores and the six key educator characteristics are presented in Table 5. It should be noted that a significant positive and moderate correlation was seen between educator age and years of educator experience, indicating a 36% overlap between these predictors. Likewise, educator age also shared a significant yet weak relationship with the predictor childcare regional site, where rural educators were more likely to be younger in age, with 12% overlap among these predictors. These overlaps indicated a considerable degree of shared variance and thus redundancy for these predictor variables.

**Table 5:** Intercorrelations between general MBQ scores and the six key educator characteristics.

**Variable**	**2**.	**3**.	**4**.	**5**.	**6**.	**7**.
1. General MBQ scores	0.20^†^	0.15	0.26^*^	0.08	−0.20^†^	0.17
2. Educator age	–	−0.05	0.60^***^	0.18^†^	0.09	0.34^**^
3. Educator role		–	0.22^*^	0.07	0.04	−0.08
4. Years of educator experience			–	0.13	0.09	0.17
5. Ever learnt instrument/sung				–	0.13	0.13
6. Highest post-school qualification					–	0.12
7. Childcare region						–

† N = 86 due to list wise deletion of cases. p < 0.10

* p < 0.05

** p < 0.01

*** *p < 0.001*.

As seen from Table 6, the only significant predictors of general beliefs about music held by early childhood and care educators were age and years of experience. In this, increased educator age and years of experience both were associated with more positive average ratings regarding general beliefs and values as to the beneficial role of music in the lives of young children. Also of note were marginally significant results in relation to the predictors of childcare regional site and highest educator qualification achieved. These findings suggested a trend for educators employed at rural (vs. urban) childcare sites and more qualified participants to express less favorable perspectives and philosophies concerning childhood music education. However, these results should be interpreted with caution as they were demonstrated to be trends only and not statistically reliable relationships.

**Table 6:** Regression analyses predicting general MBQ scores based on six key educator characteristics.

**Educator characteristic predictor(s)**	***B***	***B_***SE***_***	**β**	***R^**2**^***	***df***	***F***
**INDIVIDUAL BIVARIATE REGRESSION ANALYSES**						
Educator age (18–24, 25–34, 35–44, 45–54, 55–64, 65+ years)	0.09	0.04	0.22	0.05	1, 85	4.47^*^
Educator role (1 = general educator, 2 = higher level educator [directors and lead educators])	0.17	0.11	0.17	0.03	1, 86	2.42
Educator years of experience (0–4, 5–9, 10–14, 15–19, 20+ years)	0.11	0.04	0.28	0.08	1, 86	7.18^**^
Ever learnt instrument/sung (0 = no, 1 = yes)	0.11	0.11	0.10	0.01	1, 86	0.92
Highest post-school qualification (1 = certificate/diploma, 2 = bachelors degree, 3 = postgraduate degree)	−0.19	0.10	−0.19	0.04	1, 85	3.25
Childcare Region (1 = rural, 2 = urban)	0.24	0.13	0.20	0.04	1, 86	3.51
**STANDARD MULTIPLE REGRESSION**				0.16	6, 79	2.49^*^
Educator age	0.02	0.05	0.06			
Educator role	0.13	0.11	0.13			
Educator years of experience	0.08	0.06	0.19			
Ever learnt instrument/sung	0.06	0.11	0.05			
Highest post-school qualification	−0.25	0.10	−0.25^*^			
Childcare region	0.18	0.13	0.15			

† p < 0.10

* p < 0.05

** *p < 0.01*.

A subsequent standard multiple regression was performed with all six predictors entered simultaneously. This additional analysis was conducted to identify whether these factors held as key individual predictors in their own right, or if redundancy existed among them. Findings revealed that once the other variables had been controlled, more qualified educators reported significantly less favorable general beliefs toward the role of music in early childhood education. Given the comparatively smaller number of participants who had obtained bachelor and postgraduate degrees to certificates/diplomas, this result suggested that those with university degrees must have exhibited not only lower overall MBQ ratings, but that these less favorable viewpoints concerning music were consistent and shared for these educators rather than driven by a few outliers (as evidenced by the reduced variability within the group that facilitated the significant finding). It should be noted, however, that overall music beliefs still remained favorable for these groups i.e., all average MBQ scores sat above the neutral mid-point on the 1–7 scale for educators with bachelor and postgraduate degrees.

Not surprisingly, educator age and years of experience were no longer significant as individual predictors of overall MBQ scores, suggesting a redundancy between these variables^3^. This indicated that while only one of these two educator characteristics was required to capture adequately an increase in general music beliefs, the most logical and best choice was years of experience, as educator age displayed too much overlap with other factors in the model (which inflated its shared variance and diminished any unique contribution it may offer). Collectively, these findings revealed that while in isolation age, years of experience and qualification level shaped early childhood and care educator beliefs about the benefits of music in young children's education, only the characteristics of qualification and years of experience were required to negatively and positively predict these beliefs, respectively, due to redundancy of age with other key educator characteristics.

## Discussion

The first aim of this study was to adapt and pilot a survey of music beliefs and values for wider implementation in Australian early childhood and care settings. An existing survey, the Music Beliefs Questionnaire (MBQ; Austin and Reinhardt, 1999), was adapted for the participant group.

### The Music Beliefs Questionnaire (MBQ): Scale Factor Structure

The original Austin and Reinhardt (1999) survey had three key construct areas related to (1) aesthetic benefits, (2) quality-of-life benefits, and (3) social-emotional benefits, respectively. The proposed three-factor structure of the MBQ, modeled on this earlier work, was not better at capturing the expressed music beliefs of educators than a universal single-factor model for the current study sample. While both provided equivalently plausible explanations for the data, the MBQ was best gauged through a single average, generalized scale score, as it offered a more parsimonious account as to the underlying latent constructs.

A closer review of the original Austin and Reinhardt (1999) scale development identifies potential reasons why we did not discover the same factor structure. These researchers espoused their three-factor model following comparison and interpretation of three-, four-, and five-factor structures from their data, as suggested by a scree plot. This, along with adoption of very liberal item weights of 0.35 and above as evidence of salient factor loadings, would indicate a degree of ambiguity in identification of the underlying latent constructs. Further, they also reported a substantial reduction in Eigenvalues between their model Factor 1 and Factor 2 (i.e., a drop from 10.22 to 2.25). Based on the discontinuity principle, this could also have suggested a single-factor solution. Yet this option was overlooked in favor of a more complex multi-scale latent structure. This issue was compounded by the fact that performance of the exploratory factor analysis (EFA) to establish their initial factor structure was not followed up by a confirmatory factor analysis (CFA) with a new sample. Hence confirmation and generalizability of their chosen factor structure for the scale was not actually established. Therefore, it is difficult to have confidence that the original Austin and Reinhardt (1999) scale was indeed best represented as three distinct factors. Thus, the inability of the current study to confirm the intended three-factor model for our 37 Music Beliefs Questionnaire items, based on the previous work of Austin and Reinhardt, is unlikely to be a cause for concern.

It should be noted that four key differences were identified between the sample employed by Austin and Reinhardt (1999) that were used to claim a three-factor structure to educators' music beliefs and the sample employed in the current study that helped discover a single-factor explanation for these same beliefs. Specifically, sample characteristics differed in terms of (1) participant type, (2) country, (3) sample size, and (4) gender breakdown. While our study made use of actual educators within the early childhood education and care sector, participants within the previous study were pre-service music teachers and therefore, undergraduate university students studying music education majors. The present study was carried out in Queensland Australia, while the previous study was presumably performed in the United States of America (though this was never stated explicitly by the authors and can only be inferred). The sample size for the present research was smaller (*N* = 88) than that for the Austin and Reinhardt study (*N* = 137), where the latter exhibited a much more equal gender distribution than was shown in the current study (i.e., 77 females: 60 males vs. 87 females: 1 male). However, none of these differences in sample characteristics offers an explanation as to the difference in factor structure claimed. This may be attributed to Austin's and Reinhardt's use of unsuitably low item weights as factor loadings for the three-factor solution and disregard of the notable evidence for a single-factor model within their data (despite working with a larger, more powerful and more equal gender-distributed sample). We suggest the findings from their research offer more support to our proposed single-factor solution. We therefore conclude that a single latent factor capturing generalized beliefs about the role of music in young children's lives may offer the simplest and most suitable explanation.

### Music Education Beliefs and Values Held by Early Childhood and Care Educators

The second aim of the study was to identify the music beliefs and values held by Australian early childhood and care educators concerning music in children's learning and development.

Overall, favorable global viewpoints were expressed by all educators in the present analytic sample in relation to their beliefs about music and its value in the education of young Australian children. However, within this, degrees of favorability were shown for some specific beliefs over others. Beliefs that received the strongest support by these educators were those concerned with music offering a fun and creative vehicle for children's self-expression, enhancing their self-image, and facilitating the social inclusion of other children. However, music education was relatively less strongly endorsed for its contribution to children's education outright, or concerning the transferable skills that might help establish and cultivate other academic and self-development areas of a child's life. Therefore, it would appear that music education was most strongly and pervasively perceived as primarily an aesthetic activity for children, with less recognition, or perhaps understanding, of its utilitarian value to the broader academic and learning spheres. This finding is not surprising, with Yazejian and Peisner-Feinberg (2009) highlighting how arts activities are often placed as secondary to activities that are considered to help children in areas of cognitive development such as numeracy and literacy.

It should be acknowledged, however, that the ceiling effects observed for a large number of the Music Beliefs Questionnaire (MBQ) scale items did not allow for further discrimination between the extent of educator endorsement toward potential consequences of music education in young children's lives. This may have prevented us from further teasing apart educators' philosophical views as to the perceived outcomes of music education, including its potential significance in childhood cognitive and motor development, child psychology, and the formation and advancement of children's interpersonal skills.

### Demographic Profile of Participant Early Childhood and Care Educators, and Factors That Shape Their Beliefs and Values About Music In Education

The prototypical composition of the early childhood and care educator workforce found in the present study were young females, with a little over 6 years of experience, and who tended to work full-time in urban childcare centers. The proportion of female to male participants (87:1) may be viewed as a limitation of the study. As noted earlier, females are disproportionately represented in the early childhood education and care professions globally (Peeters et al., 2015) and the composition of this study sample reflects this global pattern. Most achieved their highest education through means of a certificate or diploma in a field relevant to the care and education of children. Very few possessed formal music qualifications, though roughly half had some personal music experience through having sung in a choir or played a musical instrument previously. However, only approximately one-sixth of the overall educator group had demonstrated continued active musical engagement, while the other one-third with musical experience had withdrawn their involvement over the years.

The standard educator profile, combined with the individual music belief item endorsements and the key educator characteristic of experience as a predictor of music beliefs, is highly informative. Specifically, it is possible to speculate that less experienced educators—who also make up the bulk of the current workforce—may hold less favorable beliefs about music because they themselves have not been educated systematically on the evidence concerning likely benefits and how best to utilize music as a tool in their teaching arsenal. Within the current early childhood education and care training programs that have been implemented in recent years in Australia, there is limited time allocated to teaching educators about the potential benefits of effective musical experiences within the classroom (0–17 h according to Letts, 2015), with little if any focus on how to use it to enhance other core academic areas for children. This may explain higher beliefs within the current educator cohort that music is primarily an aesthetic and creative device in the classroom, and relatively lower beliefs that skills gained from music can be translated to promote and accomplish academic outcomes.

This “knowledge deficit” is receiving increasing attention within the music education literature. Repeated studies reiterate the importance of improving teacher training by (re)integrating music in the teaching curriculum in order to attempt to close the gap and provide educators with a strong foundational knowledge base that draws on an increasingly rich evidence base regarding the use and role of music within early childhood education and development (Kim and Kemple, 2011; Reynolds and Burton, 2017). Corroborating this very point, Kim and Choy (2008) sought to examine whether pre-service teacher's knowledge, skills and attitudes toward music and music education improved following the completion of a specific music education course. Statistically significant improvements were shown for pre-service teachers' knowledge of musical concepts and confidence for teaching musical concepts, suggesting a correlation between knowledge and confidence—a finding echoed in other literature concerning generalist Primary teachers and music (cf. Seddon and Biasutti, 2008). Similarly, a new study of music teacher identity in Singapore found that the development of music teacher identity is highly contextualized in terms of personal biography in music, professional teacher identity and opportunities to experience successful music education practice *in situ* (Chua, 2018). Findings such as these highlight the “missing component” (see below) that exists within pre-service teacher education and warrants urgent attention.

In order to maximize the potential strengths, frameworks of practice, and learning in and through music for early childhood service providers, a shared language between the different contributory disciplines needs to exist. This would allow for a shared understanding and identification of essential characteristics of practice that support and encourage optimal child health, education and well-being. For this type of systemic change to take place, it has been argued that appropriate pre-service education is the “missing component” (Grant et al., 2018). For example, Kim and Kemple (2011) report that there is a clear positive correlation between a practitioner's musical knowledge and their beliefs concerning music's value. In their study of pre-service teachers, four domains were identified as influencing participants' beliefs: personal music experiences, experience of music in the field (as teaching), teacher training coursework, and their self-efficacy in implementing music activities in the classroom. Formative experiences as teachers can cast a long shadow on teachers' willingness to engage with and use music in the classroom.

Interestingly, aside from years of educator experience, the only other characteristic shown to shape the general music beliefs held by these childhood and care educators was qualification level. Specifically, more qualified educators tended to hold less favorable beliefs regarding the role of music education in early childhood. Although this finding was unexpected, we speculate it may reflect a rise in part from current emphases in teacher education. That is, more qualified educators are likely to have spent more of their professional preparation engaged with more formal childhood and childcare education knowledge and techniques. As a consequence, they are more likely to put greater trust in more traditional and familiar academic methods of teaching that, customarily, have not provided experience in music education as a strategy to promote child learning and engagement.

The wider implications of this can be evidenced in Russell-Bowie (2009) cross-cultural study which reported that, across five countries, Australian students gave a significantly lower rating than peers from other countries in relation to how much priority schools should place on music; suggesting an overall lower belief in music education within Australian schools. In that study, the most highly rated barriers to implementing music in the primary school classroom included factors such as “lack of priority for music” and “lack of personal musical experiences.” The authors concluded that in order for music to be a central tenet of education, teachers need to feel confident and competent in the act of teaching, learning, and making music themselves. Only then will we see change.

The picture presented above is cause for concern, as there is an implied neglect of a curriculum area (music) in our children's education and development due to teacher's not feeling confident (or convinced) about music's place and value. There is evident need to ensure that pre-service teacher education builds teachers' confidence and capacity to provide the full range of curriculum areas, and draw on the benefits of music for children's learning and development.

Knowledge of the overall early childhood and care educator profile can be used to our advantage to inform potential interventions and likelihood of intervention success. Although the stereotypical educator worked in an urban area, the rural areas cannot be neglected. This is especially true in light of the trend for rural educators to express relatively less favorable beliefs concerning the role of music in young children's education. This may stem from a lack of appropriate allocated resources and training provided to rural centers and staff for music programs, especially those that highlight and support the positive outcomes that music can create in terms of academic, developmental, and psychological advancement. It may also stem from having a greater priority on an academic “core.” Therefore, construction and availability of online resources regarding the value of music in the nurturing of young individuals' overall academic achievement and well-being may be beneficial in the uptake of music education to promote more favorable beliefs. These resources should include not only the most appropriate techniques for implementation, but also supply an explanation as to the research findings that underpin arguments for the implementation of music in children's learning and development. Such information is likely to engender greater support and uptake from potential educators, as users are more inclined to employ techniques they feel they understand, which have been communicated clearly, and for which the underlying mechanisms have been made transparent. Furthermore, provision of such repositories may help to further address and bridge the divide between educators' personal music experiences and knowledge and their professional understanding of music's use in early childhood education and development. Echoing this sentiment, Reynolds and Burton (2017) provided a list of recommendations to policy makers suggesting a stronger emphasis on the provision of better training and resources to early childhood teachers in relation to how and why we should be considering musically inclusive classrooms, in an effort to close the current gap that exists between early childhood education provision and music.

### Concluding Remarks

The aims of this study were 2-fold: first to adapt and pilot a survey of music beliefs and values for wider implementation in childcare settings; and second, to identify those musical beliefs and values held by early childhood and care educators concerning music in children's learning. As indicated above, the survey was successful in capturing these beliefs. However, the analysis suggested that a single latent generalized factor may offer the best explanation of educators' beliefs and values over the previously-conceived three-factor structure account.

Findings indicated that educators' beliefs and values on all items were above the mid-point, indicating overall positive attitudes toward music. It is noteworthy that this was despite the majority not having formal qualifications in music (98%) less than half having previously learnt a musical instrument and/or sung in a choir (48%) and only 16% currently playing a musical instrument or singing on a regular basis. It should be noted that greater years of teaching experience correlated positively with more positive attitudes, perhaps reflecting a greater emphasis on music education in these educators initial training. As noted in the literature above, for those educators who completed their training in recent years there has been limited opportunity to develop music knowledge and skills.

Approximately 20% of the sample reported graduate qualifications (Bachelor degree and above). This group reported relatively less favorable beliefs and values toward music. This finding may well-reflect the recent structuring to graduate qualifications of workforce training in the early childhood and care sector in which music education has played a lessor role. As noted above, the gender profile of the study participants might be viewed as a limitation. Further research might investigate the perspectives of male early childhood and care educators.

Cumulatively, the current study findings indicate that there is enormous potential within this population for further professional learning and development targeted at music and its conceivable wider benefits in young children's learning and lives. Given the growing evidence pool concerning the importance of music in children's lives in the home setting (Williams et al., 2015), better training programs, government and curriculum policies, and advocacy are warranted in order to challenge “old” assumptions and integrate “new” music education and development knowledge.

## Ethics Statement

This project was approved by the Institutional Human Research Ethics approval committee (approval notice 2013001040) on July 20, 2015. Written informed consent was obtained from all participants in the study.

## Author Contributions

MB designed the study, oversaw data generation, led the article draft, and revision. LF generated data in the field, undertook literature review and contributed to the article draft, and revision. JB undertook the statistical analysis and contributed to the article draft and revision. GW co-designed the study and contributed to the article draft and revision.

### Conflict of Interest Statement

There are no perceived conflicts of interest for any of the research team in relation to this article submission and the research which it reports. It should be noted that GW is an editor of the Research Topic “The impact of music on human development and well-being,” to which this article belongs. The remaining authors declare that the research was conducted in the absence of any commercial or financial relationships that could be construed as a potential conflict of interest.

## Footnotes

^1^Ten Centre Directors participated from the 7 day care centers, reflecting personnel changes during data collection.

^2^In light of the inadequate absolute, incremental, and residual fit indices displayed by the three- and one-factor models tested, we decided it prudent also to perform an exploratory factor analysis (EFA). This data-driven approach allowed the information scores gathered from participants to guide the best factor structure, which may illuminate more suitable models previously unconsidered. The EFA was conducted on the 37 MBQ items using principle axis factoring (PAF) extraction and oblique direct oblimin rotation methods, employing a delta value of 0. These choices acknowledged the measurement error inherent in self-report surveys and – since all underlying constructs were conceptualized to form facets regarding beliefs about music – permitted these extracted factors to be correlated to the extent shown in the data. Findings from the scree plot also suggested a single-factor solution based upon the principle of discontinuity. This indicated a clear steep descent in the slope of the line (and thus discontinuous ‘break') from Factor 1 to Factor 2, with a corresponding dramatic decrease in Eigenvalues from 14.52 to 2.30, respectively. Thus a one-factor structure for the MBQ items was also supported by the EFA results.

^3^Calculation of variance inflation factors (VIFs) revealed that although educator age and years of experience produced considerably higher values than the other model predictors (confirming a high degree of shared variance and thus redundancy for these variables), no multicollinearity was present in the SMR model, *VIF*_*age*_ = 1.82, *VIF*_*experience*_ = 1.73, *VIF*_*site*_ = 1.16, *VIF*_*role*_ = 1.12, *VIF*_*qualification*_ = 1.03, *VIF*_*musichistory*_ = 1.06 (where values of 1.00 indicate no shared variance between a given predictor and other model predictors, and values above 5.00 indicate multicollinearity). Further testing through SMR models run without each of educator age and years of experience, in turn, supported the notion of redundancy. Specifically, when educator age was excluded from the overall model, years of experience became a significant positive individual predictor of overall MBQ scores, whereby increased years of educator experience predicted more positive general music beliefs after the other variables had been controlled, β = 0.22, *p* = 0.042. This was in addition to the previously identified significant negative effect of highest educator qualification achieved, where more qualified participants expressed less favorable viewpoints concerning childhood music education, β = −0.25, *p* = 0.017. The remaining predictors of educator role, childcare regional site and history of ever learning a musical instrument/singing remained non-significant, βs < 0.17, *p*s > 0.127. However, when years of educator experience was excluded from the SMR model, age did not become a significant individual predictor of participants' MBQ scores once the other predictors had been controlled, β = 0.17, *p* = 0.125. Yet, again, the previously recognized significant negative influence of highest educator qualification achieved held, β = −0.25, *p* = 0.022, and the remaining predictors were non-significant as unique contributors to the model, βs < 0.18, *p*s > 0.097. It should be noted that, as stated earlier, educator age also displayed a considerable correlation and thus degree of overlap with childcare regional site as well as with years of experience. This likely explained the inability of age to achieve significance as a unique individual predictor in the SMR, as the vast majority of variance it contributed to the explanation of MBQ ratings was shared with other variables in the model. Therefore, the combined results here supported the idea that the high level of shared variance—and thus redundancy—between predictors within the model prevented educator years of experience and age emerging as significant individual predictors in the original SMR model, rather than the respective bivariate regression results with these predictors being indicative of Type I errors. This being said, it should be acknowledged that these findings speak more strongly to the redundancy of educator age over years of experience, due to the considerable overlap the former also displayed with childcare regional site, which resulted in it not demonstrating significance as an individual predictor within this additional SMR analysis.
