# Supplementary material for: Beliefs and Values About Music in Early Childhood Education and Care: Perspectives From Practitioners
Source: Front Psychol. 2019 Apr 24;10:724. doi: 10.3389/fpsyg.2019.00724 (PMC6492529; doi:10.3389/fpsyg.2019.00724)
Supplement: Supplementary file 1 [file Data_Sheet_1.pdf]

Music Beliefs in Early Childhood Education and Care Questionnaire

Directions: *For the following questionnaire, the term "**music education**" is used broadly and encompasses both child-led and educator-led music making and listening within the early childhood setting. Please read each statement carefully and place a tick '√' in the appropriate box to indicate the extent to which you personally believe the statement is true or valid.*

Please tick (√) one box only for each statement	***Very untrue*** of what I believe	***Untrue*** of what I believe	***Somewhat untrue*** of what I believe	***Neutral***	***Somewhat true*** of what I believe	***True*** of what I believe	***Very true*** of what I believe
1. Music education helps children develop and improve their motor-coordination skills	O1	O2	O3	O4	O5	O6	O7
2. Music education provides children with opportunities to improve their self-esteem	O1	O2	O3	O4	O5	O6	O7
3. Music education offers a way to include children from diverse cultures	O1	O2	O3	O4	O5	O6	O7
4. Music education supports children to learn to control their behaviour	O1	O2	O3	O4	O5	O6	O7
5. Music education encourages children's understanding of different symbol systems	O1	O2	O3	O4	O5	O6	O7
6. Music education helps children to learn about and understand emotions	O1	O2	O3	O4	O5	O6	O7
7. Music education enables children to make meaning of their experiences of the world	O1	O2	O3	O4	O5	O6	O7
8. Music education supports children's use of alternative forms of communication	O1	O2	O3	O4	O5	O6	O7
9. Music education enhances children's awareness and understanding of the arts	O1	O2	O3	O4	O5	O6	O7
10. Music education helps to develop children's self-confidence	O1	O2	O3	O4	O5	O6	O7
11. Music education helps children learn in other content areas (e.g. early literacy, numeracy)	O1	O2	O3	O4	O5	O6	O7
12. Music education increases children's awareness of other cultures	O1	O2	O3	O4	O5	O6	O7
13. Music education provides children with new ideas and skills that can be used in their play	O1	O2	O3	O4	O5	O6	O7
14. Music education enables children to develop their musical ability	O1	O2	O3	O4	O5	O6	O7
15. Music education provides children with a means of self-expression	O1	O2	O3	O4	O5	O6	O7
16. Music education encourages children to be creative	O1	O2	O3	O4	O5	O6	O7
17. Music education encourages children to participate in home and community music making	O1	O2	O3	O4	O5	O6	O7

Please tick (√) <u>one box only</u> for each statement	***Very untrue*** of what I believe	***Untrue*** of what I believe	***Somewhat untrue*** of what I believe	***Neutral***	***Somewhat true*** of what I believe	***True*** of what I believe	***Very true*** of what I believe
18. Music education is valuable in itself and needs no other justification	O1	O2	O3	O4	O5	O6	O7
19. Music education enables children to improve the quality of their lives	O1	O2	O3	O4	O5	O6	O7
20. Music education helps children develop relationships with others	O1	O2	O3	O4	O5	O6	O7
21. Music education supports the development of a child's identity	O1	O2	O3	O4	O5	O6	O7
22. Music education helps children to appreciate and understand the role of music in their culture	O1	O2	O3	O4	O5	O6	O7
23. Music education teaches children how to work together as a team	O1	O2	O3	O4	O5	O6	O7
24. Music education increases the satisfaction that children are able to derive from music	O1	O2	O3	O4	O5	O6	O7
25. Music education helps develop children's ability to focus their attention	O1	O2	O3	O4	O5	O6	O7
26. Music education helps children to develop social skills	O1	O2	O3	O4	O5	O6	O7
27. Music education allows children to have fun	O1	O2	O3	O4	O5	O6	O7
28. Music education is an important part of a holistic approach to education	O1	O2	O3	O4	O5	O6	O7
29. Music education helps children develop problem-solving skills	O1	O2	O3	O4	O5	O6	O7
30. Music education helps children to persist with challenging tasks	O1	O2	O3	O4	O5	O6	O7
31. Music education encourages children to use their imagination	O1	O2	O3	O4	O5	O6	O7
32. Music education enhances the physical well-being of children	O1	O2	O3	O4	O5	O6	O7
33. Music education enables children to understand more sophisticated and complex music	O1	O2	O3	O4	O5	O6	O7
34. Music education provides children with access to a different form of intelligence or way of knowing	O1	O2	O3	O4	O5	O6	O7
35. Music education supports children's skills in managing their own emotions	O1	O2	O3	O4	O5	O6	O7
36. Music education offers a way to include children with special learning needs	O1	O2	O3	O4	O5	O6	O7
37. Music education offers a way to include children who sometimes have trouble playing in a group with other children	O1	O2	O3	O4	O5	O6	O7
